# Efficacy and safety of capecitabine-based first-line chemotherapy in advanced or metastatic breast cancer: a meta-analysis of randomised controlled trials

**DOI:** 10.18632/oncotarget.5460

**Published:** 2015-09-30

**Authors:** Weijiao Yin, Guangsheng Pei, Gang Liu, Li Huang, Shegan Gao, Xiaoshan Feng

**Affiliations:** ^1^ Department of Oncology, the First Affiliated Hospital of Henan University of Science and Technology, Luoyang, PR, China; ^2^ Department of Respiratory Medicine, the First Affiliated Hospital of Henan University of Science and Technology, Luoyang, PR, China; ^3^ Department of Gynecology, the First Affiliated Hospital of Henan University of Science and Technology, Luoyang, PR, China

**Keywords:** capecitabine, breast cancer, first-line, meta-analysis

## Abstract

We sought to evaluate the efficacy and safety of capecitabine-based therapy as first-line chemotherapy in advanced breast cancer. Randomised controlled trials of capecitabine monotherapy or combined treatment were included in the meta-analysis. PubMed, EMBASE, the Cochrane Library database and important meeting summaries were searched systematically. Outcomes were progression-free survival (PFS), overall survival (OS), overall response rate (ORR) and grades 3–4 drug-related adverse events.

Nine trials with 1798 patients were included. The results indicated a significant improvement with capecitabine-based chemotherapy compared with capecitabine-free chemotherapy in ORR (relative risk [RR] 1.14, 95% confidence interval [CI] 1.03 to 1.26, *P* = 0.013) and PFS (hazard ratio [HR] 0.77, 95% CI 0.69 to 0.87, *P* < 0.0001). Overall survival favoured capecitabine-based chemotherapy, but this was not significant. There were more incidences of neutropenia and neutropenic fever in the capecitabine-free chemotherapy group and more vomiting, diarrhoea and hand–foot syndrome in the capecitabine-based chemotherapy group. There were no significant differences in nausea, fatigue, cardiotoxicity or mucositis/stomatitis between the two treatment regimens.

Capecitabine-based chemotherapy significantly improves ORR and PFS in patients with advanced breast cancer, but has no demonstrable impact on OS. Capecitabine-based regimens are suitable as first-line treatment for patients with advanced breast cancer.

## INTRODUCTION

Breast cancer remains the most common cancer among women worldwide. Despite significant improvements in survival outcomes over the past two decades, breast cancer remains the leading cause of cancer death in developing countries and the second in developed countries [1, 2]. The main goals of treatment of advanced breast cancer are to optimise length and quality of life. Approximately 16–20% of women with breast cancer have advanced or metastatic disease, and 50% of early stage breast cancers ultimately develop into metastatic breast cancer [1]. It is reported that advanced breast cancer contributes significantly to cancer mortality among women. Patients with advanced breast cancer do not have the option of surgical cure. Even so, the use of chemotherapy after surgery results in a significant reduction in systemic recurrence in patients with isolated loco-regional recurrences [3]. Anthracycline and taxane-based regimens are standard chemotherapy for advanced breast cancer or metastatic breast cancer. However, anthracycline-induced cardiotoxicity limits its application in widespread clinical practice [4].

Capecitabine is an oral pro-drug of 5′-deoxy-5-fluorouridineat. It shows strong anti-tumour activity in tumour cells and is well tolerated. Capecitabine has been approved as combination chemotherapy or monotherapy for the treatment of patients with locally advanced or metastatic breast cancer after failure of anthracycline- and taxane-based chemotherapy [5–7]. A series of clinical studies demonstrated that capecitabine improved overall survival and response rates in first-line chemotherapy against advanced breast cancer [8, 9]. The aim of this current analysis is to evaluate the efficacy and safety of capecitabine-based chemotherapy as first-line treatment in advanced breast cancer or metastatic breast cancer.

## RESULTS

**Figure 1:**
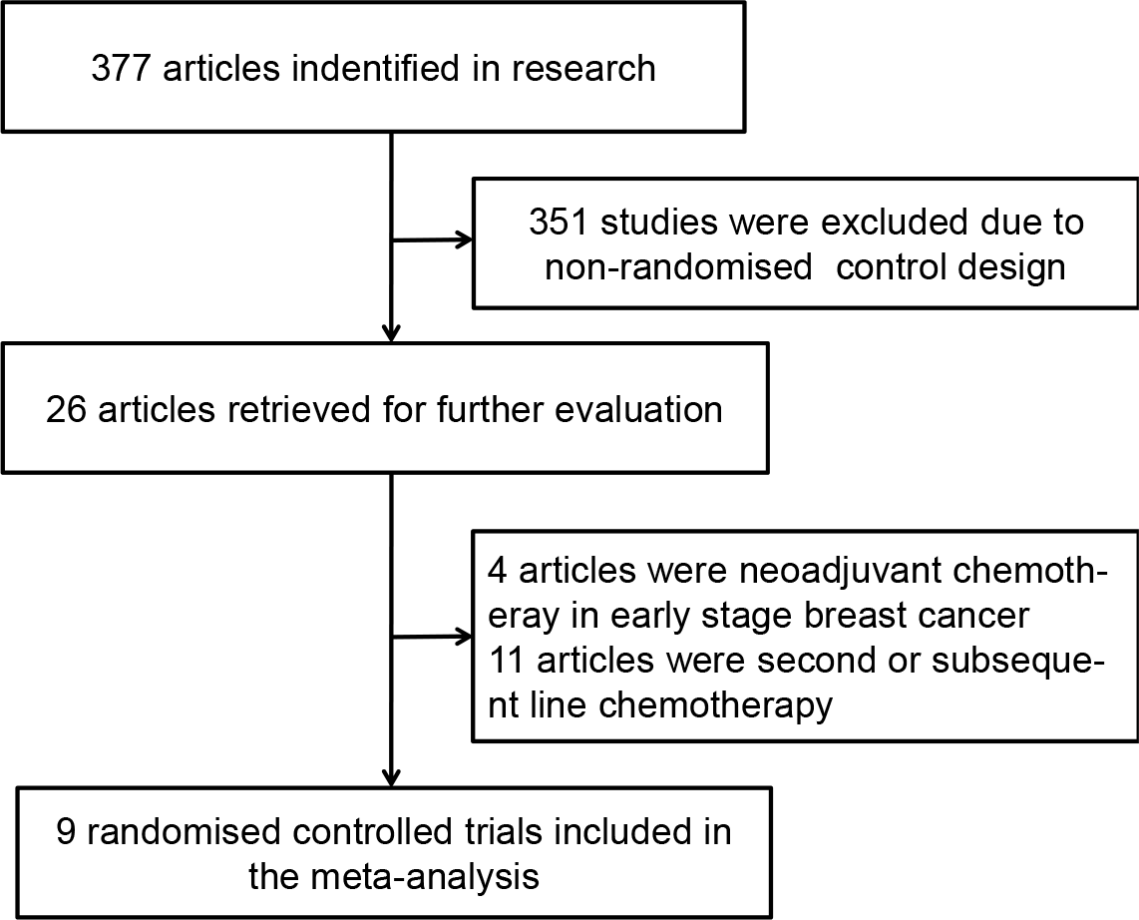
Flow diagram of the process of selecting randomised controlled trials

A total of 377 articles related to the analysis were found from the literature and subjected to the selection process (Figure 1). Finally, nine randomised controlled trials with 1798 patients met the inclusion criteria and were included in the meta-analysis [8, 9, 12–18]. The baseline characteristics of the included studies are listed in Table 1. Of the nine articles, six were of capecitabine combination therapy and three were of *capecitabine* monotherapy versus other chemotherapy.

**Table 1:** The main characteristics of RCTs included in the meta-analysis

Author	Year	Trail phase	Treatment regimens	No. of patients	Median follow-up duration
Smorenburg CH	2014	III	CAP	38	39.0 months
			PLD	40	
Lam SW	2014	II	TAX+BEV+CAP	156	41.2 months
			TAX+BEV	156	
Lück HJ	2013	III	TAX+CAP	169	24.9 months
			TAX+EPI	170	
Vici P	2011	II	DOC+CAP	36	Not stated
			DOC+GEM	36	
Stockler MR	2011	III	CAP	216	39.6 mouths
			CMF	109	
Bachelot T	2011	III	DOC+CAP	33	42.0 months
			DOC+EPI	35	
Wardley AM	2010	II	TRA+DOC+CAP	113	24.0 months
			TRA+DOC	112	
Mavroudis D	2010	III	DOC+CAP	145	43.8 months
			DOC+EPI	141	
O'Shaughnessy JA	2001	II	CAP	61	Not stated
			CMF	32	

Abbreviations: RCTs, randomised controlled tirals; CAP, capecitabine; PLD, pegylated

liposomal doxorubicin; TAX, paclitaxel; BEV, bevacizumab; EPI, epirubicin; DOC, docetaxel; GEM, gemcitabine; CMF, cyclophosphamide, methotrexate and fluorouracil

### Overall response rate

**Figure 2:**
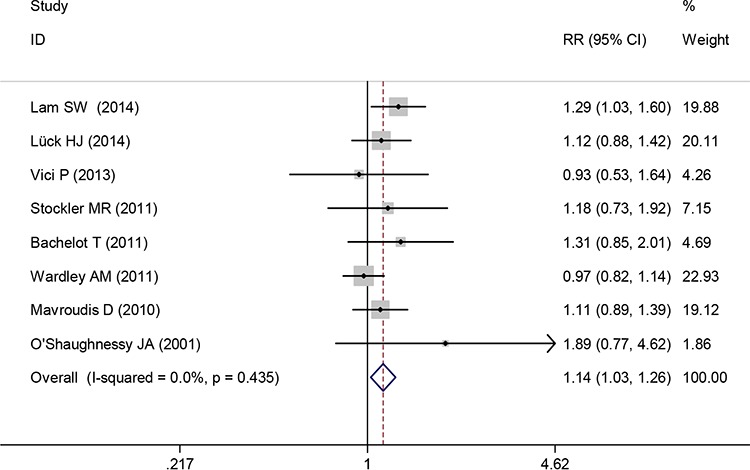
Overall response rate of capecitabine-based chemotherapy versus capecitabine-free chemotherapy

Eight trials reported the outcome of ORR and 1692 patients were included in the analysis. The pooled analysis of ORR showed that there was a significant improvement with capecitabine-based chemotherapy compared with capecitabine-free chemotherapy in the treatment of advanced or metastatic breast cancer (RR 1.14, 95% CI 1.03 to 1.26, *P* = 0.013) (Figure 2). A fixed-effect model was used because no significant heterogeneity was found between the trials (I^2^ = 0.0%, *P* = 0.435).

### Progression-free survival

**Figure 3:**
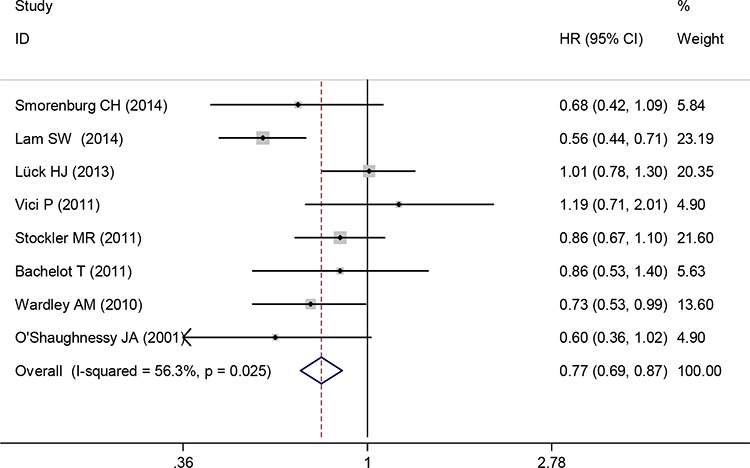
Progression-free survival of capecitabine-based chemotherapy versus capecitabine-free chemotherapy

Data for PFS were available from eight trials. The pooled HR for PFS demonstrated that capecitabine-based chemotherapy was associated with significantly longer PFS when compared with capecitabine-free chemotherapy as first-line treatment for the patients with advanced or metastatic breast cancer (HR 0.77, 95% CI 0.69 to 0.87, *P* < 0.0001) (Figure 3). A random-effects model was used because significant heterogeneity was found between the trials (I^2^ = 56.3%, *P* = 0.025).

### Overall survival

**Figure 4:**
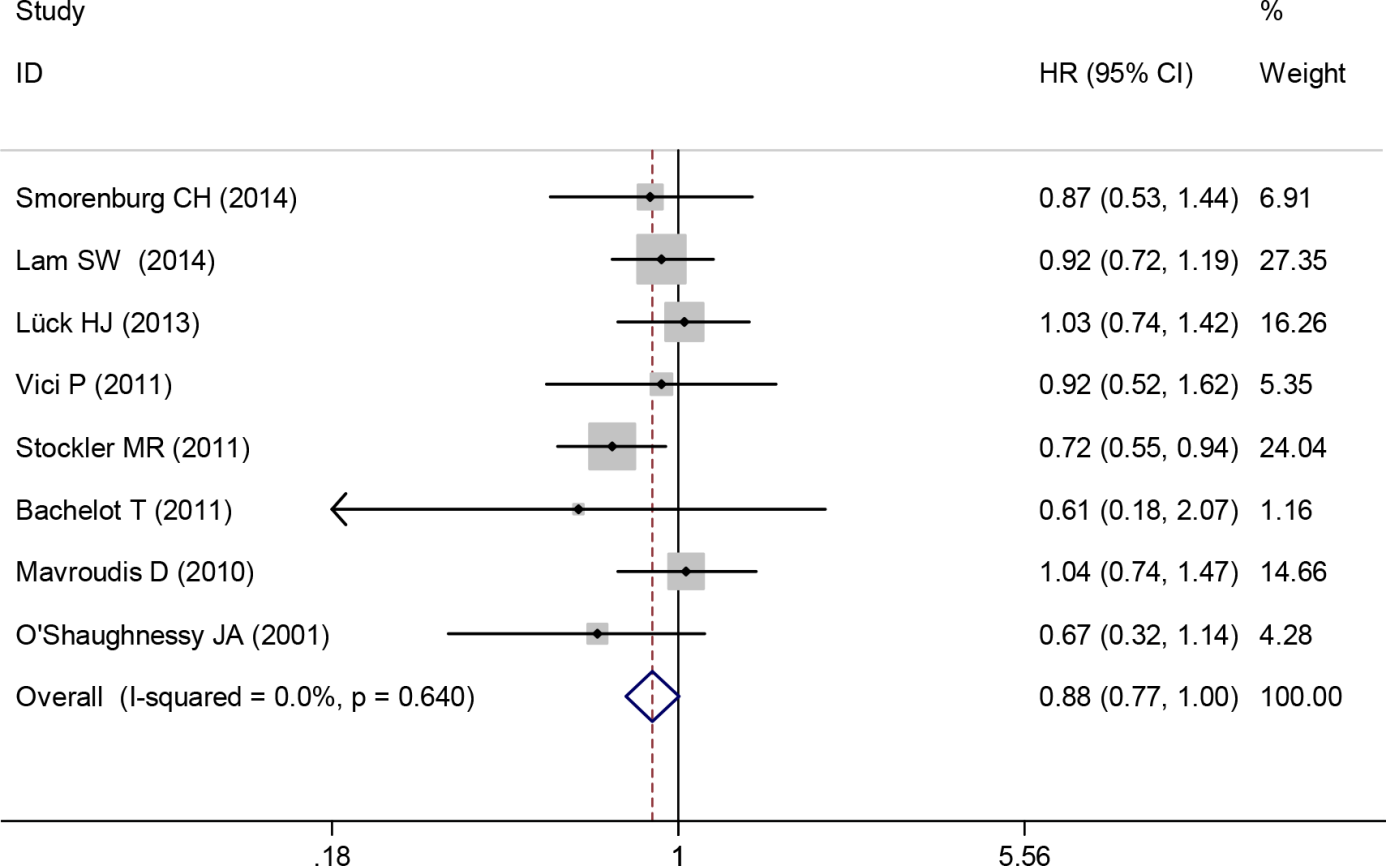
Overall survival of capecitabine-based chemotherapy versus capecitabine-free chemotherapy

Eight trials reported the HR for OS of advanced or metastatic breast cancer. Capecitabine-based therapy did not show a significant advantage over capecitabine-free chemotherapy. The pooled HR indicated that there was no significant difference in OS between the groups of capecitabine-based chemotherapy and capecitabine-free chemotherapy (HR 0.88, 95% CI 0.77 to 1.00, *P* = 0.056) (Figure 4). A fixed-effects model was used because heterogeneity between trials was not significant (I^2^ = 0.0%, *P* = 0.640).

### Safety

Common drug-related adverse events were reported in all included trials. The majority were mild (grade 1) or moderate (grade 2) in severity. The focus of our analysis is grade 3 or 4 adverse events, which are listed in Table 2. Incidences of neutropenia and neutropenic fever were fewer with capecitabine-based chemotherapy compared with capecitabine-free chemotherapy (RR 0.59, 95% CI 0.39 to 0.89, *P* = 0.012; RR 0.50, 95% CI 0.35 to 0.70, *P* < 0.0001, respectively). Incidences of anaemia and thrombocytopenia were not significantly different between the two groups. When comparing non-haematological adverse events, significantly more grade 3–4 vomiting, diarrhoea and hand–foot syndrome occurred in the capecitabine-based chemotherapy group (RR 4.47, 95% CI 2.21 to 9.03, *P* < 0.0001; RR 2.86, 95% CI 1.75 4.68, *P* = 0.0001; RR 12.4, 95% CI 3.6 to 42.8, *P* < 0.0001, respectively). However, there were no statistically significant differences in nausea, fatigue, cardiotoxicity or mucositis/stomatitis between the two arms.

**Table 2:** Outcomes of grade 3 or 4 drug-related adverse events for capecitabine-based chemotherapy versus capecitabine-free chemotherapy

Adverse events	Trials	RR and 95%CI	*P* value	Heterogeneity	
				I2	*P* value
Anemia	7	0.86(0.46–1.60)	0.641	0	0.475
Neutropenia	8	0.59(0.39–0.89)	0.012	86.3	<0.0001
Thrombocytopenia	7	0.95(0.48–1.88)	0.878	0	0.889
Neutropenic fever	7	0.50(0.35–0.70)	<0.0001	38.3	0.137
Nausea	8	1.54(0.77–3.11)	0.225	0	0.953
Vomiting	7	4.47(2.21–9.03)	<0.0001	45.7	0.101
Diarrhea	9	2.86(1.75–4.68)	0	0	0.656
Fatigue	6	0.96(0.59–1.57)	0.883	21.7	0.271
Cardiotoxicity	6	1.36(0.86–2.18)	0.2	0	0.715
Hand-foot syndrome	9	12.4(3.6–42.8)	<0.0001	57.2	0.017
Mucositis/stomatitis	9	1.02(0.31–3.34)	0.976	59.3	0.022

### Publication bias

Begg's and Egger's tests were used to assess publication bias. No publication bias was found for PFS, OS or ORR (*P* = 0.804, *P* = 0.804 and *P* = 0.216, respectively).

## DISCUSSION

Most clinical trials of first-line chemotherapy for advanced or metastatic breast cancer focus on intensive regimens. According to current guidelines, anthracycline- and taxane-based regimens are the standard primary chemotherapy for advanced or metastatic breast cancer [19, 20]. However, intensive regimes maybe unsuitable for older women or patients who have significant complications. Capecitabine-based chemotherapy used as a treatment strategy in patients pre-treated with anthracycline or taxanes in general. According the guidelines of the European School of Oncology and the European Society for Medical Oncology (ESO-ESMO) 2014 [4], capecitabine was recommended as an option for the first-line treatment of advanced or metastatic breast cancer.

Our study's pooled analysis indicates that capecitabine-based chemotherapy is significantly superior to capecitabine-free chemotherapy in terms of ORR and PFS, but that OS is similar between the two groups. This meta-analysis summarises the current randomised controlled trial evidence of the potential benefit of capecitabine-based chemotherapy as first-line treatment for the patients with advanced or metastatic breast cancer. The effects could be explained by capecitabine's synergistic effect with cytotoxic drugs through increased thymidine phosphorylase levels in tumours [20].

Most of the trials included in this review used a dose of capecitabine of 1,000 mg/m^2^ twice daily (on days 1–14 of a 3-week cycle), which is lower than that approved by the USA Food and Drug Administration (1,250 mg/m^2^ twice daily). This lower dose has been shown to lead to better tolerability of the drug without compromising its efficacy [21]. Lower haematological toxicity and higher gastrointestinal toxicity were seen in patients receiving capecitabine-based chemotherapy. It is worth noting that the rate of hand–foot syndrome toxicity was significantly higher in the capecitabine-based group. In the OMEGA study involving female patients aged ≥ 65 years, capecitabine showed acceptable tolerance as first-line chemotherapy, even in vulnerable patients and those aged ≥ 75 years [22]. A number of studies are also investigated novel dosing schedules of capecitabine. A 4-week schedule (days 1–21, every 28 days) of capecitabine-based therapy is widely used in Japan, and the ‘7/7’ regimen (7 days of therapy followed by 7 days of rest) is common in the United States [23–25]. Study has shown that apecitabine-based patients were significantly more likely to continue therapy beyond 6 months and 12 months than classical cyclophosphamide, methotrexate, and fluorouracil (CMF regimen) patients [26].

Approximately 20% of patients with metastatic breast cancer have human epidermal growth factor receptor 2 (HER-2)-positive tumours. These patients typically have a poor prognosis, with shortened PFS and OS usually [17, 27]. Trastuzumab combined with docetaxel is a standard first-line chemotherapy regime for HER-2 positive metastatic breast cancer. One trial included in this meta-analysis compared capecitabine plus trastuzumab and docetaxel with docetaxel plus trastuzumab in HER-2 positive metastatic breast cancer. A high ORR was observed between both treatment groups and PFS was significantly longer in the capecitabine-based arm [17]. Another trial demonstrated that capecitabine combined with trastuzumab improved time to progression and ORR significantly when compared with capecitabine monotherapy in patients with progression after trastuzumab-based therapy [28]. Therefore capecitabine combination therapy may be a useful treatment option for patients with HER-2 positive metastatic breast cancer, although more evidence is needed.

Several limitations need to be considered when interpreting our analysis. First, all trials except one [16] involved patients with HER-2 negative tumours. We were not able to do a subgroup analysis by HER-2 status because of the lack of sufficient data. Second, the control regimens varied widely between included trials and this may have influenced the results. Third, the sample size is relatively small. A study with a large sample size is needed in the future to better understand the role of capecitabine-based regimens in the first-line treatment of advanced breast cancer.

In summary, this analysis indicates that capecitabine-based regimens produce superior ORRs and prolong the PFS of patients with advanced or metastatic breast cancer. In addition, it has been shown that tolerability can be improved through adjusting the dosing regimen of capecitabine. Therefore, capecitabine-based chemotherapy could be a preferable option as first-line therapy for patients with advanced or metastatic breast cancer. Further research is necessary to confirm these findings.

## MATERIALS AND METHODS

### Search strategy

Two investigators (YWJ and PGSH) independently carried out a systematic search from January 1998 to May 2015, using PubMed, EMBASE and the Cochrane Library database. We also reviewed the meeting abstracts of the American Society of Clinical Oncology (ASCO) and the European Society for Medical Oncology (ESMO) for the last 10 years. Searches were limited to human clinical trials published in English. The key words were capecitabine, Xeloda, breast cancer, breast neoplasm and first-line.

### Selection criteria

Eligible studies had to meet the following inclusion criteria: randomised controlled trials involving patients with advanced or metastatic breast cancer; studies comparing capecitabine-based chemotherapy versus other chemotherapy as first-line treatment.

### Data extraction

Two investigators (YWJ and GSG) extracted data independently, using a prepared information form. Any disagreement was discussed and resolved by consensus in a meeting with a third investigator. The following data were extracted from included studies: authors, publication year, study phase, number of intention-to-treat patients, treatment regimens, randomisation method, and the results of ORR, *PFS*, OS and adverse events. We contacted the corresponding authors to obtain additional information that was not reported in the articles. The quality of the randomised controlled trials included in the meta-analysis was evaluated using the Jadad scoring system using the measures of method of randomisation, double-blinding procedure, method of allocation concealment, and withdrawals [10].

### Statistical analysis

The analysis was carried out using Stata 12.0 software, on an intention-to-treat basis. We calculated the HR for PFS and OS. The relative risk was used to calculate the ORRs and grade 3 and 4 drug-related adverse events. The HRs and their 95% CIs were obtained from the articles directly, and we extracted data from the Kaplan-Meier survival curve as reported by Parmar et al. [11] if the value was not given in the article. The heterogeneity of study outcomes was assessed by the χ^2^ test or I^2^ statistics. Heterogeneity was considered statistically significant when the *P* value was < 0.05 or I^2^ was > 50%. If significant heterogeneity existed, data was analysed using a random-effects model; otherwise, a fixed-effects model was used.
